# Effects of Repeated Contrast Therapy on Forearm Microcirculatory and Neuromechanical Recovery After Climbing-Specific Fatigue in Amateur Climbers: A Randomized Controlled Trial

**DOI:** 10.3390/jcm15134970

**Published:** 2026-06-25

**Authors:** Magdalena Hagner-Derengowska, Bartłomiej Kacprzak, Anna Michalska, Agnieszka Połaniecka, Carla Gonçalves, Robert Trybulski

**Affiliations:** 1 Sport Research Center, Faculty of Earth Sciences and Spatial Management, Nicolaus Copernicus University in Toruń, 87-100 Toruń, Poland; 2 BK21 Concept Medical Center, 90-349 Łódź, Poland; 3 Physical Education Team, Sports Research Centre, Faculty of Earth Sciences and Spatial Management, Nicolaus Copernicus University in Toruń, Lwowska 1, 87-100 Toruń, Poland; 4 Department of Management and Marketing, Koszalin University of Technology, 75-343 Koszalin, Poland; 5 Escola Superior de Desporto e Lazer, Instituto Politécnico de Viana do Castelo, 4960-320 Melgaço, Portugal; carlagoncalves@esdl.ipvc.pt; 6 The Sport Physical Activity and Health Research & Innovation Center, 4960-320 Melgaço, Portugal; 7 Faculty of Medicine, Katowice Business University, 40-659 Katowice, Poland; 8 Provita Żory Medical Center, 44-240 Żory, Poland

**Keywords:** contrast therapy, sport climbing, forearm fatigue, microcirculation, muscle stiffness

## Abstract

**Objective**: To determine whether contrast therapy improves recovery after climbing-specific forearm fatigue in amateur climbers. **Methods**: In a randomized repeated-measures trial, 40 climbers were allocated to passive recovery (n = 20) or Game Ready contrast therapy (n = 20). Both groups completed a fixed-task intermittent fingerboard protocol on a 20 mm edge using a half-crimp grip, with 7 s of work and 3 s of rest for five sets; the load was not individualized to climbing-specific maximal finger-flexor force. The intervention group received bilateral forearm treatment consisting of alternating 1 min cold (3 °C) and heat (45 °C) phases combined with pneumatic compression ranging from 15 to 75 mmHg. Sessions lasted 20 min and were administered immediately after post-fatigue testing, at 24 h and 48 h, and then three times weekly on alternate days for 8 weeks, for a total of 27 sessions. Outcomes were assessed at baseline, immediately after fatigue, at 24 h and 48 h, and after 8 weeks. Outcomes included perfusion, reactive hyperemia, stiffness, pressure pain threshold, grip strength, perceived recovery, creatine kinase, and interleukin-6. **Results**: Immediate post-fatigue responses were comparable. Contrast therapy produced greater 24 h and 48 h resting perfusion responses (+7.28 percentage points, 95% CI 6.58 to 7.98; +7.62, 95% CI 6.94 to 8.31; both adjusted *p* < 0.001). At week 8, peak hyperemic perfusion improved more with contrast therapy (+6.21 PU, 95% CI 5.62 to 6.79; *p* < 0.001). Recovery favored contrast therapy for stiffness at 48 h (−71.7 N/m, 95% CI −75.6 to −67.8), pressure pain threshold at week 8 (+8.1 N/cm^2^, 95% CI 7.3 to 8.8), and grip strength at 48 h (+7.8 kgf, 95% CI 7.3 to 8.3; all *p* < 0.001). CK and IL-6 differences were transient, and no serious adverse events or intervention-related discontinuations were recorded. **Conclusions**: Contrast therapy was associated with more favorable cutaneous perfusion, post-occlusive reactive hyperemia-derived, and neuromechanical recovery outcomes, whereas biochemical differences were limited and time-dependent. The vascular findings do not establish improved endothelial function or nitric-oxide-mediated vasodilation because these mechanisms were not directly assessed. Trial registration: ISRCTN49499065 on 23 June 2025.

## 1. Introduction

Sport climbing has developed into a highly specialized discipline in which performance depends on the interaction of maximal finger-flexor force, local endurance, technical skill, and sport-specific testing characteristics [1,2,3]. During climbing, the forearm finger flexors perform repeated sustained and intermittent isometric contractions that can compromise local muscle oxygenation and require rapid reoxygenation during brief recovery periods [4,5]. These demands are clinically relevant because overuse and acute climbing injuries are concentrated in the hand and upper limb, with finger flexor pulley injury and high-intensity crimping identified as important concerns in climbers [6,7].

Recovery after demanding exercise is not limited to restoration of perceived readiness but comprises interacting physiological, neuromuscular, and psychological processes that evolve over different time courses [8]. At the local tissue level, microcirculation is central to oxygen delivery, capillary exchange, and removal of metabolites that accumulate during intense muscle work [9]. Laser Doppler flowmetry and hyperemic challenge protocols are widely used to quantify cutaneous microvascular reactivity, but their validity depends on strict standardization of temperature, probe placement, occlusion or heating stimulus, and pretest conditions [10,11,12]. Assessment of forearm stiffness, pressure pain threshold, grip strength, and perceived fatigue can complement perfusion measures because exercise-induced muscle stress may affect tissue mechanical behavior, nociceptive sensitivity, functional capacity, and psychophysiological recovery [8,13,14].

Contrast therapy, traditionally based on alternated cold and warm exposures, has been studied as a recovery strategy after exercise-induced muscle damage and sport-specific fatigue [15,16]. Systematic reviews and meta-analyses indicate that contrast water therapy can improve selected recovery outcomes compared with passive recovery, but effects vary by outcome domain, comparator, and follow-up interval [15,16,17]. Hydrotherapy and cryotherapy evidence is heterogeneous, with recent network evidence suggesting differential effects across creatine kinase, soreness, and neuromuscular recovery rather than a uniform recovery benefit [17]. Cold exposure effects also appear dose-dependent, as systematic evidence indicates that immersion temperature and duration influence recovery responses after exercise-induced muscle damage [18,19].

Evidence on device-based contrast or cryo-compression is more limited but directly relevant to the present intervention because these protocols combine thermal stimuli with intermittent pneumatic pressure rather than water immersion alone [20,21]. Recent forearm studies in combat-sport athletes show that cryo-compression, cold-heat contrast pressure therapy, and compression contrast therapy can acutely alter tissue perfusion, muscle stiffness or tone, pressure pain threshold, and strength-related outcomes [20,22,23]. However, translation of these findings to climbing remains uncertain because climbing involves unique finger-flexor loading, small-hold grip mechanics, and repeated intermittent contractions that differ from combat-sport forearm demands [4,5,24]. Climbing-specific studies have examined forearm shaking and active recovery, cold-water immersion, compression sleeves, and the time course of recovery after competition [1,3,7,24]. However, this evidence has predominantly concerned short between-bout recovery, isolated finger-flexor performance, or observational recovery trajectories rather than repeated thermal-compression treatment assessed across vascular, neuromechanical, functional, perceptual, and biochemical domains. Existing climbing research therefore provides a physiological and practical foundation for the present study but does not establish whether repeated contrast therapy improves recovery over 24–48 h or produces longer-term changes after repeated exposures [1,3,7,24].

Previous climbing studies have examined short between-bout recovery, forearm shaking, cold-water immersion, compression sleeves, and post-competition recovery trajectories. These studies show that local oxygenation, finger-flexor performance, pain, and perceived readiness do not necessarily recover in parallel. What remains unclear is whether a repeated thermal-compression intervention can modify both the early 24–48 h recovery course and the response observed after several weeks of exposure [8,10,15,20]. The present study aimed to evaluate the effects of repeated Game Ready contrast therapy using alternating heat, cold compression, and pneumatic pressure on microvascular responses, muscle stiffness, pressure pain threshold, maximal hand grip force, subjective recovery indices, and selected biochemical markers in amateur sport climbers after a climbing-specific fatigue protocol and after an 8-week intervention period. We hypothesized that contrast therapy would improve microvascular reactivity and reduce forearm muscle stiffness compared with passive recovery while producing more limited effects on long-term biochemical markers than on vascular and neuromechanical outcomes [15,20].

## 2. Materials and Methods

### 2.1. Study Design

**Figure 1:**
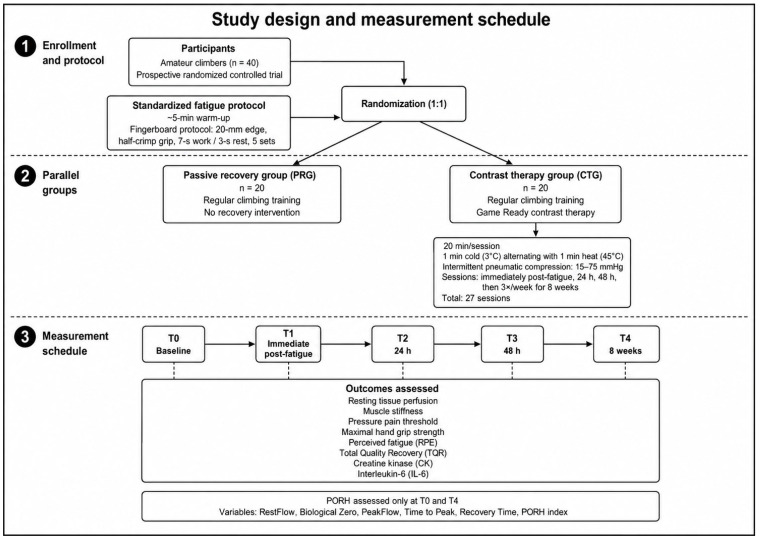
Study design, intervention structure, and measurement schedule. Fixed-task fingerboard fatigue protocol: 20 mm edge, half-crimp grip, 7 s work/3 s rest, five sets; relative intensity not individualized.

This prospective, randomized, controlled, two-arm interventional trial with repeated measurements was designed to evaluate the acute and adaptive effects of contrast therapy on forearm recovery responses in amateur sport climbers (Figure 1). The reporting structure was organized according to the CONSORT 2025 statement for randomized trials [25]. The study compared a passive recovery group (PRG) with a contrast therapy group (CTG) after a standardized climbing-specific forearm fatigue protocol and during an 8-week intervention period.

Participants were allocated in a 1:1 ratio to PRG or CTG. Both groups completed the same fatigue protocol, testing schedule, laboratory procedures, and follow-up assessments. The CTG received repeated Game Ready contrast therapy consisting of alternating cold and heat exposure combined with intermittent pneumatic compression, whereas the PRG received passive rest and was asked to avoid additional recovery procedures. The principal outcome domains were microcirculatory response, post-occlusive reactive hyperemia, muscle stiffness, pressure pain threshold, maximal hand grip strength, perceived fatigue and recovery, and selected biochemical markers of exercise-induced fatigue and recovery.

### 2.2. Ethics, Registration, and Setting

The study was conducted at the Provita Medical Center in Zory, Poland, under standardized laboratory conditions. The protocol was approved on 11 June 2025 by the Ethics Committee for Scientific Research of Physiotherapists at the Polish Physiotherapy Association (Resolution No. 1.06.2025) and was registered in the ISRCTN registry under the identifier ISRCTN49499065 on 23 June 2025. All participants received written and verbal information about the purpose, procedures, potential risks, and withdrawal rights before enrollment and provided written informed consent. The study was conducted in accordance with the Declaration of Helsinki and applicable ethical principles for human-participant research.

All testing sessions were performed in the morning between 09:00 and 12:00 to reduce circadian variability in microcirculatory, neuromuscular, and biochemical measures. Ambient temperature was maintained at 21–22 °C, and relative humidity was maintained between 45% and 55%. Participants were asked to refrain from strenuous exercise, alcohol, ergogenic beverages, and unreported recovery procedures before testing sessions. Before measurements, participants rested quietly in a seated position for at least 10 min to stabilize hemodynamic and microcirculatory responses.

### 2.3. Participants

**Figure 2:**
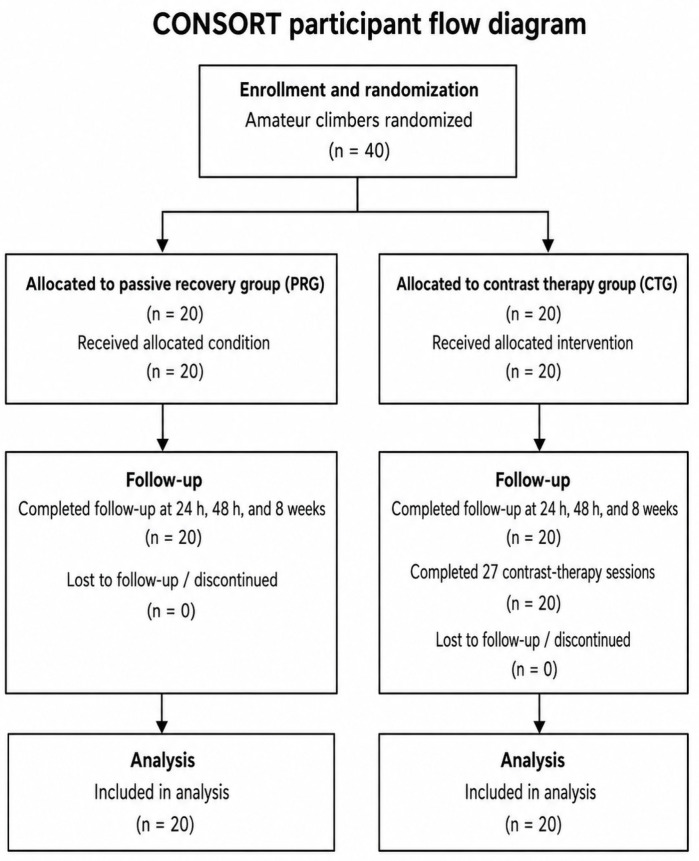
Consort participant flow diagram.

Forty-four volunteers were recruited and expressed their willingness to participate in the study. However, four were excluded before enrolment because they had sustained musculoskeletal injuries during the four weeks preceding the study. Forty healthy amateur climbers were enrolled and randomized, with 20 participants assigned to PRG and 20 assigned to CTG (Figure 2). The PRG included 13 men and 7 women, and the CTG included 12 men and 8 women. Participants were 22 to 38 years of age and had at least 2 years of climbing experience. Eligibility required regular recreational or sport climbing training at least twice weekly, the ability to complete the standardized fatigue protocol, and the absence of current musculoskeletal injury that would restrict hand, wrist, forearm, elbow, or shoulder function. The dominant upper limb was recorded at baseline and used for all functional, biomechanical, nociceptive, and microcirculatory assessments.

Exclusion criteria were resting blood pressure greater than 140/90 mmHg before testing, abnormal vascular screening findings, current treatment for upper-limb injury; recent injury of the hand, wrist, forearm, or elbow; skin lesions or dermatological disease at the measurement site; myofascial abnormalities that could interfere with measurement validity; tattoos at the laser Doppler assessment site; infection; fever; excessive fatigue; use of analgesic, anti-inflammatory, or vasoactive medications; and inability to safely complete any study procedure. Participants were screened before inclusion using systolic and diastolic blood pressure, resting heart rate, ankle–brachial index, and individual arterial occlusion pressure assessed near the radial artery. Only participants with normal screening values, including ankle–brachial index values between 0.99 and 1.09 and no evidence of peripheral vascular disorder, continued to the experimental phase.

### 2.4. Standardized Fatigue Protocol

**Figure 3:**
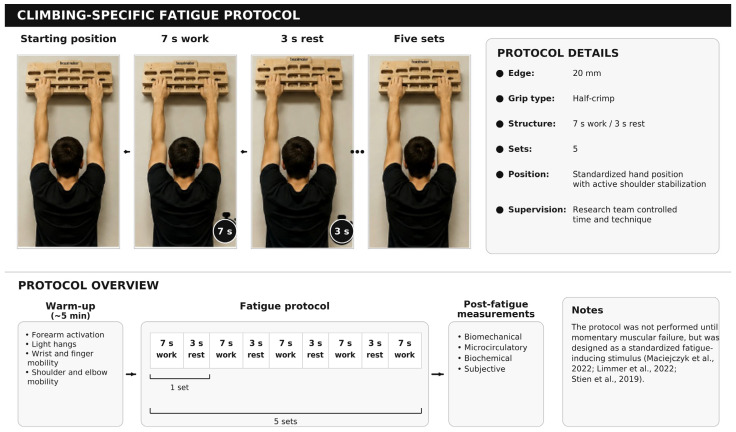
Climbing-specific fatigue protocol. Participants performed standardized intermittent isometric hangs on a 20 mm edge using a half-crimp grip. The protocol used a 7 s work and 3 s rest structure for five sets and standardized the task configuration rather than the relative intensity. Participants supported their own body mass, and the load was not adjusted to a common percentage of climbing-specific maximal finger-flexor force. The protocol was intended to induce a reproducible forearm-loading response and was not continued to momentary muscular failure.

Before the fatigue protocol (Figure 3), participants performed a standardized warm-up of approximately 5 min consisting of forearm and hand activation, repetitive squeezing of a soft rehabilitation ball, light hanging exercises, dynamic wrist and finger flexion-extension, and brief shoulder-girdle and elbow mobility exercises [26]. The main fatigue protocol was performed on a 20 mm fingerboard edge using a half-crimp grip. Participants completed intermittent isometric hangs using a 7 s work and 3 s rest structure for five sets. Because participants suspended their own body mass, the absolute external load varied according to body mass. No added or counterbalanced load was used to prescribe the task at a common percentage of climbing-specific maximal finger-flexor force. Accordingly, edge depth, grip position, duty cycle, set number, and body position were standardized, whereas relative exercise intensity was not individualized. The protocol was designed as a fixed-task fatigue-inducing stimulus and was not continued to momentary muscular failure [5,24,27]. Participants maintained a standardized hanging position with active shoulder-girdle stabilization and controlled upper-limb alignment. A member of the research team supervised all repetitions, rest intervals, execution technique, and participant safety. Biomechanical, microcirculatory, biochemical, functional, and subjective measurements were initiated immediately after completion of the fatigue protocol according to the predefined testing sequence.

### 2.5. Intervention and Comparator

The contrast therapy protocol was developed based on previous studies investigating the effects of cold compression and contrast therapy on muscle biomechanical properties, tissue perfusion, and post-exercise recovery in athletes [22,28]. Before the main experimental phase, all participants assigned to the contrast therapy group (CTG) underwent a familiarization session 7 days before study initiation. The familiarization procedure consisted of a sham Game Ready intervention to familiarize participants with the thermal and compression stimuli and minimize novelty-related effects during the intervention period. The familiarization session was developed in accordance with previously published Game Ready protocols. The intervention was performed using the Game Ready system (Avanos Medical, Alpharetta, GA, USA). Contrast therapy was applied bilaterally to the forearm muscles using pneumatic compression cuffs. The intervention consisted of alternating cold and heat stimulation combined with intermittent pneumatic compression. The protocol was based on previous studies investigating cold compression and contrast therapy in sports recovery.

The therapy consisted of alternating 1 min cold stimulation at 3 °C and 1 min heat stimulation at 45 °C. During the intervention, pneumatic compression alternated between 15 and 75 mmHg. The total duration of one therapy session was 20 min. The decision to use a 20 min protocol was based on previous findings investigating the optimal duration of cold and heat compression therapy and its effects on tissue perfusion, muscle stiffness, and recovery-related biomechanical properties [23]. The first contrast therapy session was applied immediately after the post-fatigue measurements. Additional sessions were performed 24 h and 48 h after the fatigue protocol. Subsequently, participants in the CTG continued the intervention for 8 weeks, performing the therapy three times weekly on alternate days. In total, each participant completed 27 recovery sessions, including the acute post-exercise sessions and the long-term intervention phase. Participants assigned to the CTG used only the recovery protocol included in the study and were instructed not to use any additional recovery methods during the intervention period. The control condition was defined as the passive recovery group (PRG). Participants assigned to the PRG did not receive any recovery intervention other than passive rest. They maintained their regular climbing training routines while refraining from cold compression therapy, massage, sauna, compression garments, physiotherapeutic recovery procedures, or any additional regeneration methods throughout the 8-week intervention period. Compliance with the protocol was monitored during weekly contact with the research team.

All sessions were administered under direct supervision by trained research personnel. Before each session, participants were asked about new illness, injury, medication use, skin symptoms, altered sensation, or other conditions that could affect treatment safety. The skin beneath and adjacent to the cuffs was inspected before and immediately after treatment for persistent erythema, pallor, cyanosis, swelling, abrasion, blistering, or other evidence of pressure- or temperature-related irritation. During treatment, participants were instructed to report excessive pain, burning, numbness, tingling, intolerable cold or heat, dizziness, nausea, diaphoresis, visual disturbance, presyncope, or any other unexpected symptom.

### 2.6. Measurement Schedule and Outcome Domains

Measurements were obtained at five predefined time points: T0, baseline before the fatigue protocol; T1, immediately after the fatigue protocol; T2, 24 h after the fatigue protocol; T3, 48 h after the fatigue protocol; and T4, after completion of the 8-week intervention period. Resting perfusion, muscle stiffness, pressure pain threshold, maximal hand grip strength, perceived fatigue, perceived recovery, CK, and IL-6 were assessed according to the repeated-measures schedule. The complete PORH assessment was performed only at T0 and T4 to evaluate longer-term vascular adaptation rather than immediate acute recovery. Primary outcome domains were microcirculatory response, muscle stiffness, and forearm muscle function. Secondary outcome domains were pressure pain threshold, subjective fatigue and recovery, and biochemical markers associated with acute exercise-induced fatigue and recovery.

All assessments were performed on the dominant upper limb unless the procedure required bilateral intervention delivery. Measurement sites were identified using anatomical landmarks at the first session and marked to improve reproducibility across repeated assessments. Measurements were performed by experienced physiotherapists trained in microcirculatory, biomechanical, nociceptive, and functional assessment procedures. The order of testing was kept constant across visits to reduce order-related measurement variability.

#### 2.6.1. Microcirculatory Assessment and Post-Occlusive Reactive Hyperemia

Microcirculatory assessment was performed using laser Doppler flowmetry with a PeriFlux System device (Perimed AB, Järfälla, Sweden) and a standard fiber-optic contact probe for cutaneous perfusion assessment. The probe was positioned on the distal pulp of the index finger of the dominant upper limb. Signal acquisition and processing were performed with PeriSoft software (version 5.6.2, Perimed AB, Järfälla, Sweden). Before each measurement session, the instrument was calibrated according to the manufacturer’s instructions using the supplied calibration motility solution. Participants were seated in a relaxed position with the dominant forearm supported, the elbow flexed at approximately 60°, and movement minimized throughout signal acquisition.

The microcirculatory protocol included resting tissue perfusion and post-occlusive reactive hyperemia (PORH). Resting tissue perfusion was recorded as an acute microvascular outcome following the fatigue protocol and recovery intervention. For PORH, individual arterial occlusion pressure was determined before the test and used to standardize the ischemic stimulus [29]. A 10 cm pneumatic occlusion cuff was placed proximally on the upper arm and inflated to the participant-specific 100% arterial occlusion pressure for 5 min. Immediately after cuff release, continuous perfusion recording was used to characterize the reactive hyperemic response. Variables extracted from the PORH recording were resting flow expressed in perfusion units, biological zero during complete arterial occlusion expressed in perfusion units, peak flow after cuff release expressed in perfusion units, time to peak expressed in seconds, recovery time expressed in seconds, and the PORH index calculated as peak flow divided by resting flow. Resting flow was interpreted as an acute microvascular response, whereas the complete PORH profile was used to characterize the magnitude and temporal pattern of the cutaneous perfusion response to standardized transient ischemia at baseline and week 8. PORH was not treated as a direct measure of nitric oxide bioavailability, nitric oxide synthase activity, or endothelial function [30].

#### 2.6.2. Muscle Stiffness Assessment

**Figure 4:**
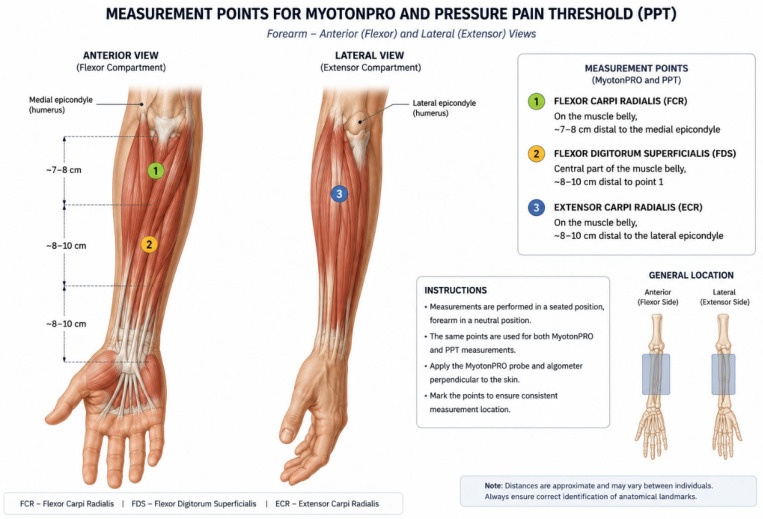
Anatomical locations for muscle stiffness and pressure pain threshold assessment. Point 1 corresponded to flexor carpi radialis, point 2 to flexor digitorum superficialis, and point 3 to extensor carpi radialis. The same marked sites were used for myotonometric and algometric measurements across all repeated assessments.

Forearm muscle stiffness was assessed using the MyotonPRO device (Myoton AS, Tallinn, Estonia, 2021). The device delivers a brief mechanical impulse perpendicular to the skin surface and records the damped oscillatory response of the underlying tissue, from which stiffness is calculated [31]. Only stiffness was selected for analysis because it was the most relevant Myoton-derived parameter for post-exercise mechanical recovery in the present protocol [32]. Measurements were performed at three standardized anatomical points on the dominant forearm (Figure 4): flexor carpi radialis, flexor digitorum superficialis, and extensor carpi radialis. For each site, five consecutive impulses were applied, and the mean value was used for analysis. Muscle stiffness was expressed in Newtons per meter.

#### 2.6.3. Pressure Pain Threshold

**Figure 5:**
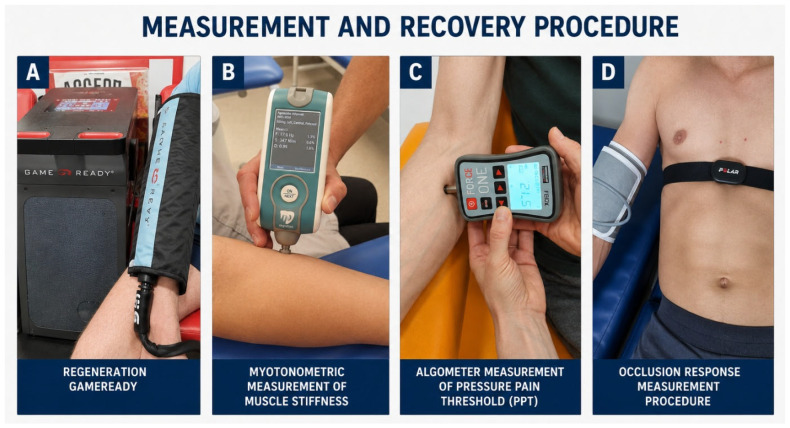
Measurement and recovery procedures. Panel (**A**) shows the Game Ready forearm recovery procedure; panel (**B**) shows myotonometric assessment of muscle stiffness; panel (**C**) shows digital algometry for pressure pain threshold assessment; and panel (**D**) shows the occlusion procedure used for vascular response assessment.

Pressure pain threshold was assessed using a digital algometer FPIX 25 (Wagner Instruments, Greenwich, CT, USA, 2021). Measurements were performed at the same three anatomical points used for muscle stiffness assessment. The participant was seated with the dominant forearm supported and the elbow flexed at approximately 60°. A 4 mm probe was placed perpendicular to the skin surface, and pressure was increased gradually until the participant verbally indicated the transition from pressure sensation to pain (Figure 5). Three consecutive trials were performed at each site with a short recovery interval between trials. The mean of the three trials was used for analysis, and the pressure pain threshold was expressed in Newtons per square centimeter.

#### 2.6.4. Hand Grip Strength Assessment

Maximal hand grip strength was assessed as maximal isometric forearm force using an electronic hand dynamometer and expressed in kilogram force. Before testing, participants completed ten maximal squeezes of a soft ball followed by a 10 s forearm stretch to standardize neuromuscular preparation. Testing was performed on the dominant upper limb with the participant standing and the tested arm positioned comfortably alongside the body. Participants were instructed to perform a maximal voluntary hand grip contraction for 5 s. Three trials were performed with 30 s of rest between attempts, and the mean of the three trials was used as the final value.

#### 2.6.5. Subjective Fatigue and Recovery Assessment

Subjective fatigue was assessed using the Borg rating of perceived exertion scale, scored from 0 to 10 [33], with higher values indicating greater perceived fatigue or exertion. Recovery status was assessed using the Total Quality Recovery scale, a single-item global rating originally developed to quantify an athlete’s perceived recovery status. The TQR scale ranges from 6 to 20 points, with higher values indicating better perceived recovery and readiness. The original conceptual basis of the TQR is described by Kenttä and Hassmén [34].

More direct reliability evidence is available from national-level swimmers, in whom same-day TQR test–retest assessment produced a typical-error coefficient of variation of 3.8% (90% confidence limits, 2.9–5.5%) and a signal-to-noise ratio of 1.8 for week-to-week variation [35]. However, no climbing-specific TQR reliability or validity study was identified, and a COSMIN-based systematic review [36] concluded that the measurement properties of commonly used single-item athlete-reported monitoring measures remain insufficiently established. Accordingly, TQR was treated in the present study as a secondary subjective recovery indicator rather than as a fully validated climbing-specific instrument.

Internal consistency coefficients were not calculated because TQR contains only one item. Test–retest reliability was also not estimated in the present trial because duplicate ratings were not obtained under a condition in which participants’ true recovery status could reasonably be assumed to remain stable. Before the beginning of the study, all participants were familiarized with both scales and received standardized instructions regarding the meaning of the scale anchors and response procedures. Both scales were completed under quiet laboratory conditions after completion of the biomechanical and functional assessments at each measurement session.

#### 2.6.6. Biochemical Assessment

Biochemical assessment included serum creatine kinase activity and serum interleukin-6 concentration as markers related to exercise-induced muscle stress, inflammatory signaling, and recovery. Venous blood samples of approximately 8 mL were collected from the antecubital vein by a qualified laboratory diagnostician using sterile vacuum collection tubes. Blood sampling was performed in the morning under standardized laboratory conditions. Samples were transported immediately to the laboratory and centrifuged at 3000× *g* for 10 min at 4 °C using an MPW-54 centrifuge (MPW Med Instruments, Randox Laboratories Polska Sp. z o.o, Warsaw, Poland). Serum was separated and stored at −80 °C until analysis.

Creatine kinase activity was determined using colorimetric spectrophotometry with commercially available diagnostic reagents according to the IFCC kinetic method and was analyzed on an automated biochemical analyzer (Randox, Warsaw, Poland). Creatine kinase activity was expressed in units per liter. Interleukin-6 concentration was determined using high-sensitivity enzyme-linked immunosorbent assay kits according to the manufacturer’s instructions and measured with a BioTek microplate spectrophotometer (BioTek Instruments, Winooski, VT, USA). Interleukin-6 concentration was expressed in picograms per milliliter. Analyses were performed in duplicate by the same experienced laboratory personnel to reduce analytical variability.

#### 2.6.7. Safety Screening and Protocol Adherence

Before inclusion in the study, all participants underwent a safety screening procedure due to the application of the PORH protocol and repeated vascular occlusion procedures. The screening included assessment of systolic and diastolic blood pressure, resting heart rate, ankle–brachial index (ABI), and determination of individual arterial occlusion pressure (AOP). Only participants with normal vascular screening results and without contraindications to vascular occlusion testing were qualified for further participation. 

The determination of 100% arterial occlusion pressure was considered a critical component of the safety procedure and was used to individualize the ischemic stimulus during PORH assessment. This approach increased participant safety and improved standardization and reproducibility of vascular responses between participants by accounting for individual hemodynamic variability.

To ensure methodological consistency and protocol adherence, all participants were instructed to maintain their regular climbing training routines throughout the intervention period. Training compliance was monitored weekly using individual training logs and direct communication with the research team. Participants assigned to the contrast therapy group completed all recovery sessions under direct supervision of the research personnel at the Provita Medical Center. Attendance and adherence to the intervention protocol were monitored and documented throughout the entire 8-week study period.

Participants were excluded from the final per-protocol analysis if they completed less than 75% of the planned training volume or failed to complete the prescribed recovery intervention sessions. Additional exclusion criteria included interruption of the intervention protocol, use of additional unauthorized recovery procedures, injury during the study period, or inability to complete the experimental procedures. This methodological approach was used to ensure high protocol fidelity, minimize interventional bias, and improve the internal validity of the study. However, participation was complete in both cases, resulting in a 100% completion rate.

An adverse event was defined as any unfavorable or unintended symptom, sign, skin finding, or clinical occurrence arising during or after a study procedure, irrespective of whether it was considered causally related to the intervention. Events of particular interest for the thermal-compression intervention included local discomfort or pain, persistent erythema, skin irritation or abrasion, blistering or thermal injury, swelling, numbness or paresthesia, pressure intolerance, dizziness, nausea, presyncope, syncope, and other vasovagal symptoms. Safety data were summarized by the number of participants experiencing at least one event and by the total number of events across treatment sessions. Repeated occurrences of the same event in one participant were recorded as separate episodes when they occurred during different treatment sessions.

### 2.7. Randomization and Masking

Participants were randomly allocated in a 1:1 ratio to PRG or CTG. Because the intervention involved perceptible temperature and compression stimuli, blinding of participants and therapists was not feasible. To reduce measurement bias, the same standardized order of assessments, body positioning, anatomical landmarks, device calibration procedures, and environmental conditions were maintained at every time point. The random-sequence-generation method was employed using a randomizer online, and an allocation-concealment procedure was ensured.

### 2.8. Sample Size

A priori sample size justification: The a priori sample size was based on the primary inferential target of the trial, defined as the group × time interaction for the main microvascular recovery outcome, i.e., resting tissue perfusion, because the central hypothesis concerned whether contrast therapy modified the longitudinal recovery trajectory after climbing-specific forearm fatigue rather than producing only a single time point difference. In a single-blind randomized trial of compression contrast therapy after isometric forearm fatigue, significant group × time effects were reported for perfusion units (partial η^2^ = 0.525), pressure pain threshold (partial η^2^ = 0.159), muscle tension (partial η^2^ = 0.130), and maximal force (partial η^2^ = 0.165), corresponding to Cohen’s f values of approximately 1.05, 0.44, 0.39, and 0.45 [23].To avoid basing the trial on optimistic single-study estimates and to account for differences in population, fatigue model, follow-up duration, and intervention schedule, the calculation used a conservative small-to-moderate interaction effect of f = 0.20. The sample size was calculated in G*Power 3.1 using the F-test family, “ANOVA: repeated measures, within–between interaction,” with two groups, five repeated measurements, α = 0.05, power = 0.80, assumed average correlation among repeated measurements = 0.50, nonsphericity correction ε = 1.00, and equal 1:1 allocation, which yielded a minimum required sample of 32 participants, corresponding to 16 participants per group [37]. Because repeated-measures trials should also account prospectively for attrition, incomplete outcome acquisition, and unusable longitudinal recordings, the target sample was increased to 40 participants, corresponding to 20 participants per group, thereby preserving the minimum analyzable sample under approximately 20% loss while maintaining the prespecified power for the primary group × time interaction.

### 2.9. Statistical Procedures

Descriptive statistics are reported as mean ± standard deviation for continuous outcomes and as counts with percentages for categorical variables. The normality and homoscedasticity assumptions were evaluated using residual plots, quantile–quantile plots, Shapiro–Wilk tests of model residuals or change scores, and inspection of standardized residuals. Extreme observations were screened using standardized residuals and externally studentized residuals; values were retained unless they were both statistically influential and incompatible with the measurement protocol. Creatine kinase and interleukin-6 were analyzed on the natural-log scale for inferential testing because these biochemical variables are strictly positive and typically right-skewed after exercise. For each participant and follow-up time point, biochemical change was defined as ln(follow-up value) − ln(baseline value), which is equivalent to ln(follow-up value/baseline value). Between-group contrasts were estimated from these natural-log change scores. To facilitate interpretation on the original multiplicative scale, the estimated log contrasts and their confidence limits were back-transformed and expressed as percentage differences using 100 × [exp(estimate) − 1] [38].

Baseline demographic, anthropometric, climbing-related, and vascular screening characteristics were summarized descriptively by randomized group using mean ± standard deviation for continuous variables and counts with percentages for categorical variables. No formal hypothesis tests of baseline differences were performed. For longitudinal outcomes measured at baseline, immediately after the fatigue protocol, 24 h, 48 h, and week 8, the primary inferential estimands were between-group differences in change from the relevant baseline resting value. These planned contrasts quantify whether the contrast therapy group changed differently from the passive recovery group at each recovery time point. The same logic was applied to resting tissue perfusion, except that acute perfusion responses were expressed as percent change from the resting value within each acute testing block, separately for the initial acute session and the post-intervention week 8 session.

For the main repeated-measures questions, the analysis corresponded to a linear mixed-effects repeated-measures model with fixed effects for group, time, and the group-by-time interaction and a participant-specific random intercept. The planned contrasts from these models were reported as adjusted mean differences in change with 95% confidence intervals. Because the dataset was balanced and complete, the model-based time-specific interaction contrasts were equivalent to the observed between-group change contrasts. Post-occlusive reactive hyperemia variables, which were recorded only at baseline and week 8, were analyzed as between-group differences in pre-to-post change. The post-occlusive reactive hyperemia outcomes included resting tissue perfusion, biological zero, peak reactive hyperemic perfusion, time to peak perfusion, recovery time to baseline perfusion, and the post-occlusive reactive hyperemia index.

Multiplicity was controlled within coherent outcome families using the Holm procedure applied to prespecified contrasts within each family. The outcome families were resting tissue perfusion, muscle stiffness, pressure pain threshold, hand grip strength, perceived fatigue, perceived recovery, creatine kinase, interleukin-6, and post-occlusive reactive hyperemia. All statistical tests were two-sided, and statistical significance was defined as multiplicity-adjusted *p* < 0.05. Effect sizes for between-group differences in change were calculated as Hedges’ g with approximate 95% confidence intervals. Hedges’ g was calculated by dividing the between-group difference in individual change scores by the pooled standard deviation of the change scores and applying small-sample bias correction. For CK and IL-6, Hedges’ g was calculated using the participant-level natural-log change scores and their pooled standard deviation. Thus, the biochemical Hedges’ g estimates quantify standardized between-group separation on the same scale used for inferential testing. Hedges’ g was not back-transformed because it is a dimensionless standardized effect; the separately reported percentage contrasts provide the corresponding interpretation on the original multiplicative scale. For descriptive interpretation, the absolute magnitude of Hedges’ g was classified as trivial (<0.20), small (0.20–0.59), moderate (0.60–1.19), large (1.20–1.99), very large (2.00–3.99), or extremely large (≥4.00) [39]. The sign of Hedges’ g indicates the direction of the contrast, whereas the magnitude category was assigned using |g|. Qualitative labels refer to the point estimate and were interpreted alongside the raw between-group difference and its 95% confidence interval. They were used as descriptive aids and not as evidence, in isolation, of clinical or practical importance [40,41]. Analyses were performed using SPSS software (version 23.0, IBM, New York, NY, USA).

## 3. Results

The control group included 13 men and seven women, and the contrast therapy group included 12 men and eight women. Baseline demographic, anthropometric, climbing-related, and vascular screening characteristics were similar descriptively between groups, with no material imbalances apparent (Table 1). All ankle–brachial index values were within the predefined eligibility range. All 20 participants assigned to contrast therapy completed the prescribed sessions. No serious adverse events, treatment-related skin injuries, persistent sensory abnormalities, vasovagal episodes, or intervention discontinuations were recorded.

### 3.1. Microcirculatory Responses

**Figure 6:**
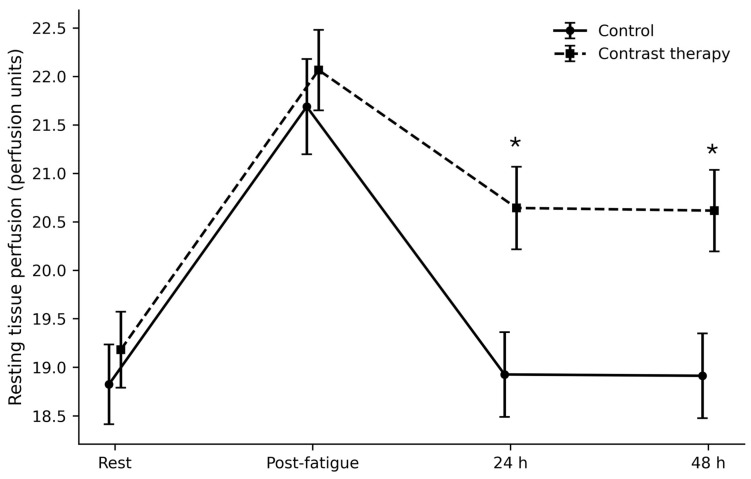
Week 8 resting tissue perfusion trajectory after the climbing-specific fatigue protocol. Values are group means with 95% confidence intervals. The passive recovery group is displayed with a solid line and circles, and the contrast therapy group is displayed with a dashed line and squares. The complete rest–post-fatigue–24 h–48 h sequence was assessed after completion of the 8-week intervention; therefore, week 8 represents the assessment occasion rather than an additional x-axis time point. PU, perfusion units. *: significantly different at *p* < 0.05.

The cutaneous post-occlusive reactive hyperemia responses assessed at baseline and week 8 differed between groups, with greater peak perfusion and faster response kinetics in the contrast therapy group. In contrast, the 24 h and 48 h post-fatigue perfusion responses were clearly separated between groups. During the initial acute session, perfusion remained close to baseline in the passive recovery group at 24 h and 48 h, whereas the contrast therapy group maintained higher perfusion relative to rest; the between-group differences in percent change were +7.28 percentage points at 24 h and +7.62 percentage points at 48 h, both multiplicity-adjusted *p* < 0.001. After the 8-week intervention, the immediate post-fatigue percent increase again did not differ between groups, but the contrast therapy group maintained higher perfusion at both 24 h and 48 h, with between-group differences of +7.07 and +7.01 percentage points, respectively, both multiplicity-adjusted *p* < 0.001. The week 8 perfusion trajectory is shown in Figure 6, and descriptive values for primary outcomes are presented in Table 2.

**Figure 7:**
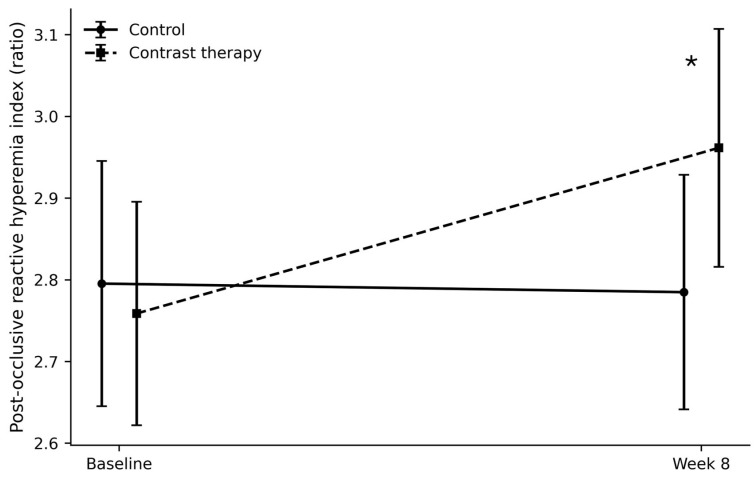
Post-occlusive reactive hyperemia index at baseline and week 8. Values are group means with 95% confidence intervals. The passive recovery group is displayed with a solid line and circles, and the contrast therapy group with a dashed line and squares. The post-occlusive reactive hyperemia index was calculated as peak reactive hyperemic perfusion divided by resting tissue perfusion. The PORH index is a descriptive measure of the cutaneous hyperemic response and does not identify a specific endothelial or vasoactive mediator. *: significantly different at *p* < 0.05.

Adaptive microvascular reactivity, assessed by the post-occlusive reactive hyperemia protocol at baseline and week 8, also favored contrast therapy. Peak reactive hyperemic perfusion increased by 6.24 PU in the contrast therapy group and by 0.03 PU in the passive recovery group, yielding a between-group difference in change of +6.21 PU (95% CI, +5.62 to +6.79; adjusted *p* < 0.001). Time to peak perfusion shortened by 5.75 s in the contrast therapy group and was essentially unchanged in the passive recovery group, resulting in a between-group difference in change of −5.65 s (95% CI, −6.19 to −5.11; adjusted *p* < 0.001). Recovery time to baseline perfusion decreased by 33.66 s with contrast therapy and did not improve in the passive recovery group, producing a between-group difference in change of −34.02 s (95% CI, −36.60 to −31.43; adjusted *p* < 0.001). The post-occlusive reactive hyperemia index increased by 0.20 in the contrast therapy group and remained stable in the passive recovery group, with a between-group difference in change of +0.21 (95% CI, +0.17 to +0.25; adjusted *p* < 0.001). Biological zero did not differ meaningfully between groups over time. Adaptive post-occlusive reactive hyperemia results are summarized in Table 3 and displayed for the hyperemic index in Figure 7. Based on the prespecified absolute Hedges’ g categories, the standardized between-group effects were extremely large for peak reactive hyperemic perfusion, time to peak, and recovery time; very large for the post-occlusive reactive hyperemia index and resting tissue perfusion; and small for biological-zero perfusion. The confidence interval for biological-zero perfusion included zero, indicating substantial uncertainty regarding the direction and magnitude of that effect.

### 3.2. Biomechanical, Nociceptive, and Functional Recovery

**Figure 8:**
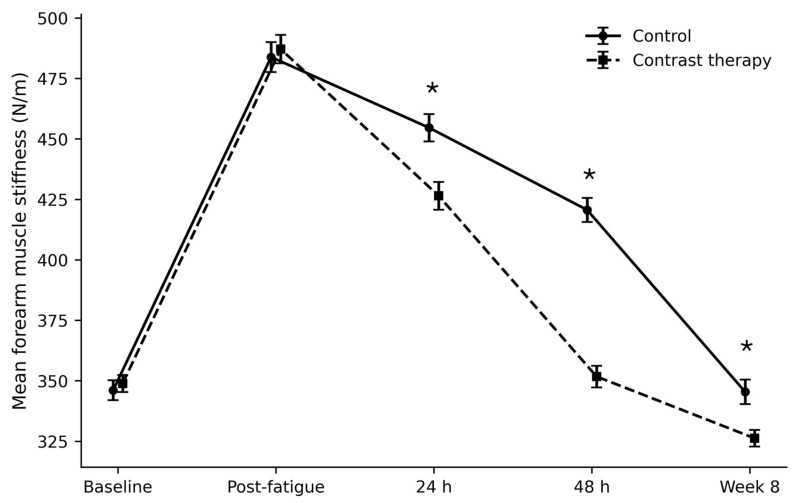
Mean forearm muscle stiffness across the intervention and recovery period. Values are group means with 95% confidence intervals. Muscle stiffness values represent participants’ averages across the flexor carpi radialis, flexor digitorum superficialis, and extensor carpi radialis measurement points. The passive recovery group is displayed with a solid line and circles, and the contrast therapy group with a dashed line and squares. *: significantly different at *p* < 0.05.

Mean forearm muscle stiffness increased substantially and similarly immediately after fatigue in both groups, indicating that the fatigue protocol produced a comparable acute mechanical response before the recovery intervention had time to act. The immediate post-fatigue increase from baseline was +137.7 ± 6.9 N/m in the passive recovery group and +138.3 ± 7.4 N/m in the contrast therapy group; the between-group difference in change was +0.5 N/m (95% CI, −4.1 to +5.1; adjusted *p* = 0.813). Thereafter, stiffness recovered more rapidly in the contrast therapy group. At 24 h, the between-group difference in change was −30.9 N/m (95% CI, −35.1 to −26.7; adjusted *p* < 0.001), and at 48 h it was −71.7 N/m (95% CI, −75.6 to −67.8; adjusted *p* < 0.001). By week 8, mean stiffness was below baseline in the contrast therapy group and near baseline in the passive recovery group, yielding a between-group difference in change of −22.0 N/m (95% CI, −23.9 to −20.0; adjusted *p* < 0.001). The group trajectories are shown in Figure 8.

**Figure 9:**
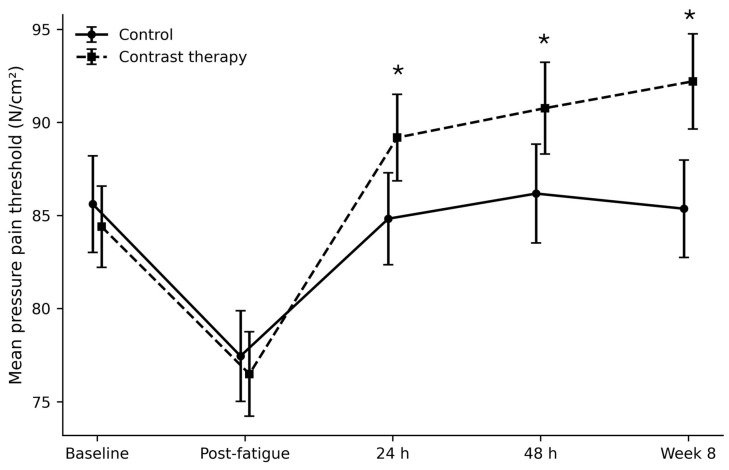
Mean pressure pain threshold across the intervention and recovery period. Values are group means with 95% confidence intervals. Pressure pain threshold values represent participants’ averages across the flexor carpi radialis, flexor digitorum superficialis, and extensor carpi radialis measurement points. The passive recovery group is displayed with a solid line and circles, and the contrast therapy group with a dashed line and squares. *: significantly different at *p* < 0.05.

Pressure pain threshold decreased immediately after the fatigue protocol to a similar extent in both groups, consistent with a comparable acute increase in mechanical pain sensitivity. The immediate post-fatigue change was −8.16 ± 1.27 N/cm^2^ in the passive recovery group and −7.90 ± 1.10 N/cm^2^ in the contrast therapy group, with no meaningful between-group difference in change (adjusted *p* = 0.495). Subsequent recovery favored contrast therapy. The between-group differences in change were +5.57 N/cm^2^ at 24 h (95% CI, +5.01 to +6.13; adjusted *p* < 0.001), +5.80 N/cm^2^ at 48 h (95% CI, +5.14 to +6.46; adjusted *p* < 0.001), and +8.06 N/cm^2^ at week 8 (95% CI, +7.34 to +8.78; adjusted *p* < 0.001). These data indicate a higher pressure pain threshold during recovery and after the intervention in the contrast therapy group, without evidence of an immediate between-group difference directly after fatigue. The pressure pain threshold trajectory is shown in Figure 9.

**Figure 10:**
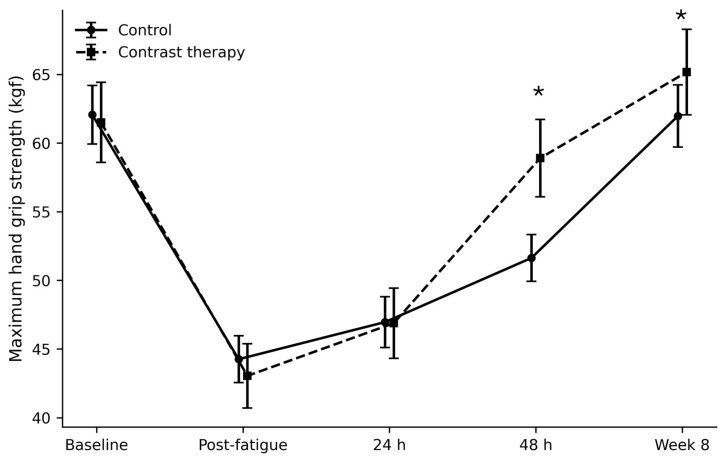
Maximum hand grip strength across the intervention and recovery period. Values are group means with 95% confidence intervals. Hand grip strength was measured on the dominant upper limb and is expressed in kilogram force. The passive recovery group is displayed with a solid line and circles, and the contrast therapy group with a dashed line and squares. *: significantly different at *p* < 0.05.

Maximum hand grip strength declined similarly in both groups immediately after fatigue and remained similarly depressed at 24 h. The immediate post-fatigue between-group difference in change was −0.65 kgf (95% CI, −2.30 to +1.00; adjusted *p* = 0.862), and the 24 h difference in change was +0.48 kgf (95% CI, −1.18 to +2.14; adjusted *p* = 0.862). By 48 h, the contrast therapy group had recovered more force than the passive recovery group, with a between-group difference in change of +7.84 kgf (95% CI, +7.33 to +8.34; adjusted *p* < 0.001). At week 8, hand grip strength was modestly above baseline in the contrast therapy group and essentially unchanged from baseline in the passive recovery group, with a between-group difference in change of +3.76 kgf (95% CI, +3.22 to +4.30; adjusted *p* < 0.001). The hand grip strength trajectory is displayed in Figure 10.

### 3.3. Perceptual and Biochemical Recovery

Perceived fatigue increased similarly immediately after the fatigue protocol in both groups, whereas later recovery favored contrast therapy. At 24 h, the contrast therapy group reported a smaller increase from baseline in perceived fatigue than the passive recovery group, with a between-group difference in change of −0.95 points (95% CI, −1.41 to −0.49; adjusted *p* < 0.001). The corresponding differences were −0.65 points at 48 h (95% CI, −1.05 to −0.25; adjusted *p* = 0.004) and −1.05 points at week 8 (95% CI, −1.46 to −0.64; adjusted *p* < 0.001). Total Quality Recovery followed a complementary pattern. The contrast therapy group showed higher recovery scores than the passive recovery group at 24 h, 48 h, and week 8, with between-group differences in change of +1.15 points (95% CI, +0.56 to +1.74; adjusted *p* < 0.001), +2.30 points (95% CI, +1.70 to +2.90; adjusted *p* < 0.001), and +2.00 points (95% CI, +1.42 to +2.58; adjusted *p* < 0.001), respectively.

Creatine kinase and interleukin-6 increased after fatigue in both groups. For creatine kinase, the immediate post-fatigue log-relative change did not differ between groups; however, the relative increase from baseline was smaller in the contrast therapy group at 24 h and 48 h. The between-group difference in log-relative change corresponded to a −6.3% relative difference at 24 h (95% CI, −6.9% to −5.8%; adjusted *p* < 0.001) and a −10.1% relative difference at 48 h (95% CI, −10.6% to −9.7%; adjusted *p* < 0.001). The corresponding Hedges’ g estimates calculated from the natural-log change scores were −7.10 (95% CI, −8.81 to −5.39) at 24 h and −13.56 (95% CI, −16.67 to −10.45) at 48 h, both classified as extremely large standardized effects. The negative direction indicates a smaller CK increase in the contrast therapy group. At week 8, creatine kinase had returned to baseline-range values in both groups, with no evidence of a between-group difference in relative change and a trivial standardized effect (g = −0.13; 95% CI, −0.75 to +0.49). Interleukin-6 showed a similar immediate post-fatigue increase in both groups. At 24 h, the contrast therapy group had a smaller relative increase from baseline than the passive recovery group, corresponding to a −24.7% between-group relative difference (95% CI, −27.5% to −21.8%; adjusted *p* < 0.001). The corresponding standardized effect calculated from the natural-log change scores was extremely large (g = −4.78; 95% CI, −6.01 to −3.54). Interleukin-6 did not differ meaningfully between groups at 48 h or week 8. The standardized effects were trivial at both 48 h (g = −0.16; 95% CI, −0.78 to +0.46) and week 8 (g = +0.13; 95% CI, −0.49 to +0.75), and both confidence intervals included zero. Planned between-group contrasts for primary, perceptual, and biochemical outcomes are provided in Table 4.

## 4. Discussion

### 4.1. Principal Findings

This randomized repeated-measures study showed that repeated contrast therapy after a climbing-specific forearm fatigue protocol was associated with a more favorable recovery profile than passive recovery across vascular, mechanical, nociceptive, functional, perceptual, and selected biochemical domains. The immediate post-fatigue responses were broadly comparable between the randomized groups, indicating similar mean group-level perturbations before the recovery trajectories diverged. However, because the fixed-task hanging protocol was not prescribed relative to climbing-specific maximal finger-flexor force, these group-level findings do not establish that all participants experienced the same relative intensity or degree of fatigue. Thereafter, the most consistent effects favored contrast therapy for maintenance of resting tissue perfusion at 24 h and 48 h, improvement in post-occlusive reactive hyperemia after 8 weeks, lower forearm muscle stiffness, higher pressure pain threshold, and improved subjective recovery. The standardized effect magnitudes were heterogeneous since immediate contrasts were predominantly trivial or small, perceptual effects ranged from moderate to very large, and several delayed objective and biochemical contrasts were extremely large. Because Hedges’ g was standardized using the pooled variability of change scores, the very large and extremely large values indicate that the between-group separation was substantial relative to the observed variability. They should not be interpreted as direct evidence of equivalent clinical importance across outcomes. Overall, the results support the primary hypothesis that repeated contrast therapy improves microvascular and neuromechanical recovery after climbing-specific forearm fatigue, while supporting the more cautious secondary hypothesis that long-term biochemical effects are limited.

The comparability of the acute post-fatigue response is important because sport climbing places high local demands on the finger flexors through repeated or sustained isometric contractions performed under conditions that can restrict local oxygen delivery [4,42]. Prior climbing studies have shown that climbing-specific finger flexor strength, endurance, and oxygenation kinetics are closely related to climbing ability, with faster reoxygenation during intermittent rest phases reported in trained climbers [4]. These findings support the physiological relevance of restoring both force production and local oxygenation after intensive climbing-specific loading [43].

Climbing-specific intermittent finger flexor tests also have a substantial aerobic contribution despite their high local force demands, emphasizing the physiological relevance of recovery between short contraction bouts [5]. In this context, the absence of immediate between-group differences reduces concern that randomization produced a systematic difference in the mean acute response between groups, but it does not exclude participant-level heterogeneity in relative intensity, internal load, or proximity to task failure. Climbing-specific endurance tests commonly prescribe force relative to a same-task maximum, and experimental hand grip evidence demonstrates that endurance time and physiological responses vary with the percentage of maximal voluntary contraction. The delayed separation in maximal hand grip strength is also coherent with the broader concept that recovery is not a single process but a time-dependent interaction among neuromuscular, metabolic, perceptual, and vascular responses [8,44].

### 4.2. Microvascular Interpretation

The clearest physiological signal involved the microvascular outcomes. Resting finger-pulp perfusion remained higher in the contrast therapy group at 24 h and 48 h, while the week-8 PORH assessment showed greater peak cutaneous perfusion, a shorter time to peak, a shorter recovery time, and a higher PORH index. These findings represent related but distinct observations: resting perfusion characterizes the post-exercise recovery period, whereas PORH characterizes the cutaneous perfusion response to standardized transient ischemia.

One possible, but unverified, explanation is that repeated exposure to alternating temperatures and pneumatic compression altered local vasomotor or hemodynamic stimuli. Heating can increase cutaneous blood flow, cooling can induce complex vasoconstrictor and cold-induced vasodilator responses, and pneumatic compression can alter venous and arterial hemodynamics [45,46]. These externally supported mechanisms establish biological plausibility but were not tested as mediators in the present trial.

The present PORH findings must not be interpreted as evidence that contrast therapy enhanced nitric oxide production, nitric oxide synthase activity, NO-dependent vasodilation, or endothelial function. The study did not measure nitric oxide or its metabolites, endothelial biomarkers, pharmacologically isolated NO-dependent dilation, acetylcholine-mediated dilation, flow-mediated dilation, or shear rate during treatment.

Moreover, cutaneous PORH is a composite response involving several vascular and neurovascular mechanisms. Human experimental studies have demonstrated an important contribution from cutaneous sensory nerves, and nitric oxide synthase inhibition has not consistently altered the cutaneous reactive hyperemic response [47,48]. Direct investigation of NO-dependent cutaneous vasodilation generally requires a protocol designed to isolate the NO contribution, such as controlled local heating combined with intradermal delivery of a nitric oxide synthase inhibitor [10]. Such procedures were not included in the present study.

The present results therefore support an association between contrast therapy and altered cutaneous perfusion and PORH kinetics, without identifying the responsible mediator, signaling pathway, or cellular target. Nitric oxide-dependent vasodilation, sensory-nerve activity, endothelium-derived hyperpolarization, thermal control of vascular smooth muscle, autonomic regulation, and compression-related pressure or shear effects remain alternative or interacting hypotheses rather than demonstrated mechanisms.

The anatomical interpretation must also remain restricted to the skin. Finger-pulp laser Doppler flowmetry measures relative cutaneous perfusion and does not directly quantify blood flow, oxygen delivery, or reoxygenation in the forearm flexor muscles. The data consequently cannot establish that the observed cutaneous response mediated the improvements in stiffness, pain threshold, hand grip strength, or perceived recovery.

### 4.3. Neuromechanical, Nociceptive, and Functional Interpretation

The stiffness and pressure-pain-threshold findings indicate that contrast therapy was associated with a more favorable recovery of tissue mechanical behavior and mechanical pain sensitivity. Both outcomes changed similarly between groups immediately after fatigue but subsequently diverged, indicating that the intervention did not abolish the initial perturbation and was more likely to have influenced processes occurring during recovery.

Several mechanisms are plausible. Cooling can reduce peripheral nerve-conduction velocity and increase the pain threshold, whereas heating can increase local blood flow and connective-tissue extensibility; external compression may influence venous emptying and interstitial-fluid movement [49,50,51]. These mechanisms were not directly assessed. The observed reduction in MyotonPRO stiffness cannot by itself distinguish altered muscle tone, passive viscoelastic behavior, intramuscular pressure, edema, or changes in surrounding connective tissue. Similarly, an increased pressure pain threshold indicates reduced mechanical pain sensitivity but does not identify whether the effect arose from peripheral nerve cooling, altered local tissue pressure, central pain modulation, expectancy, or a combination of these factors.

The delayed hand-grip-strength advantage at 48 h and week 8 may indicate that recovery of force production followed improvements in the mechanical and nociceptive environment rather than occurring immediately after treatment. Reduced stiffness or pain sensitivity could plausibly permit greater voluntary force expression, but mediation was not tested, and the present data cannot establish this causal sequence. The recovery of maximal hand grip strength by approximately 24 h after elite bouldering competition reported by Gáspari et al. [52] indicates that grip-force recovery can occur more rapidly than perceived readiness in some climbing settings. The more prolonged strength deficit observed in the passive-recovery group in the present study may reflect differences in participant level, fatigue protocol, grip configuration, total loading, and measurement procedures rather than a direct discrepancy between studies. Moreover, conventional hand grip dynamometry does not reproduce the 20 mm half-crimp position or the force-sharing requirements of climbing. The strength findings should therefore be interpreted as recovery of general maximal hand grip function rather than direct evidence of improved climbing performance.

### 4.4. Perceptual and Biochemical Findings

The perceptual outcomes were directionally concordant with several physiological and functional outcomes, with lower perceived fatigue and higher Total Quality Recovery scores in the contrast therapy group during the post-fatigue recovery period. Recovery-monitoring consensus statements emphasize that recovery is multidimensional and that subjective ratings can provide complementary information regarding an athlete’s perceived recovery state [8]. However, TQR is a single-item subjective measure, and its reliability and validity have not been specifically established in climbers. In national-level swimmers, TQR demonstrated relatively low same-day test–retest error, although the study included only a small subgroup and did not establish validity in climbing populations [35]. A subsequent COSMIN-based systematic review found that single-item athlete-reported monitoring measures were widely used but generally lacked adequate validation evidence [36]. More recent research examining other single-item recovery ratings in dancers reported acceptable test–retest reliability but only partial construct validity; this evidence supports the feasibility of single-item recovery monitoring but does not validate the specific TQR scale in climbers [53]. The present TQR findings should therefore be interpreted as supportive participant-reported evidence of perceived recovery rather than as an independently validated measure of physiological recovery. The biochemical outcomes should be interpreted more cautiously than the vascular and mechanical outcomes because serum creatine kinase is an indirect and highly variable marker of exercise-related muscle membrane disruption [14,54]. Interleukin-6 is also complex because acute exercise-induced IL-6 reflects metabolic and immune signaling rather than being a simple marker of harmful inflammation [13,55]. The present pattern, with lower relative creatine kinase at 24 h and 48 h and lower relative interleukin-6 at 24 h but no sustained week 8 biochemical separation, therefore indicates transient attenuation of selected blood-marker responses rather than broad long-term biochemical remodeling. The standardized effects calculated from the natural-log change scores were extremely large for CK at 24 h and 48 h and for IL-6 at 24 h, whereas the corresponding week 8 effects were trivial. These large standardized estimates indicate substantial short-term between-group separation relative to the low observed variability of the log-change scores. However, they should be interpreted together with the back-transformed relative difference (−6.3% and −10.1% for CK and −24.7% for IL-6) and should not be equated with clinical importance, reduced tissue injury, or sustained systemic anti-inflammatory adaptation. This interpretation is consistent with the wider recovery literature in which thermal and hydrotherapy interventions often show outcome-, dose-, and time-specific effects rather than uniform improvements across subjective, performance, and biochemical markers [13,15,16].

### 4.5. Strengths and Limitations

A strength of this study was the integration of vascular, mechanical, nociceptive, functional, perceptual, and biochemical outcomes across acute and longer-term measurement points. This allowed the recovery response to be interpreted as a time-dependent, multidomain process rather than from a single surrogate outcome. The randomized design, controlled laboratory timing, repeated anatomical measurement sites, participant-specific arterial occlusion pressure, and complete follow-up also strengthened internal consistency.

Some limitations should be considered when interpreting these findings. The contrast therapy protocol combined thermal alternation and intermittent pneumatic compression, meaning that the independent effects of cold, heat, compression, and their sequencing cannot be separated. The microvascular assessment was standardized and individualized by arterial occlusion pressure, but finger-pulp laser Doppler measurements remain a cutaneous surrogate and should not be interpreted as a direct measurement of forearm muscle perfusion. The climbing-specific fatigue protocol standardized the external task configuration but not the relative exercise intensity. Participants supported their own body mass on the same edge, and differences in body mass, climbing-specific finger strength, grip efficiency, training status, and fatigue resistance may therefore have produced different relative demands among participants. No maximal finger-flexor force test on the same 20 mm edge, load-cell recording, force–time integral, or individual time-to-task-failure measure was obtained. Consequently, the available hand grip dynamometry data cannot retrospectively establish the percentage of climbing-specific maximal force represented by the protocol for each participant. Similar mean immediate responses between the randomized groups reduce concern about systematic group-level imbalance, but they do not demonstrate equivalence of the participant-level fatigue dose. The findings should therefore be interpreted as recovery after a fixed climbing-specific loading task rather than recovery after an individually matched relative-intensity stimulus. The microvascular measurements represented finger-pulp skin perfusion rather than direct forearm-muscle perfusion, and no mechanistic measurements of muscle oxygenation, tissue temperature, edema, intramuscular pressure, nerve conduction, shear rate, or molecular vascular signaling were obtained. In particular, the study did not assess nitric oxide concentration or bioavailability, nitric oxide synthase activity, endothelial biomarkers, pharmacologically isolated NO-dependent dilation, acetylcholine-mediated microvascular dilation, or conduit-artery flow-mediated dilation. Therefore, the observed PORH differences cannot be attributed specifically to enhanced nitric-oxide-mediated vasodilation or improved endothelial function. Any proposed contribution of nitric oxide, sensory nerves, thermal vasomotor regulation, or compression-induced hemodynamic stimuli remains an unverified mechanistic hypothesis. Future research should use larger multicenter randomized designs with sham-controlled thermal-compression comparators, direct muscle oxygenation or perfusion measures, and longer follow-up after treatment cessation. Future fatigue protocols should also determine maximal finger-flexor force using the same edge and grip configuration, use added or counterbalanced load to prescribe a defined relative intensity, and record force output throughout the task to quantify the force–time integral, performance decrement, and proximity to task failure.

### 4.6. Practical and Clinical Significance

The principal practical implication concerns the 24–48 h period after demanding forearm loading. In amateur climbers who train on consecutive or closely spaced days, a recovery strategy that is associated with lower stiffness and pain sensitivity, maintained cutaneous perfusion, and later recovery of hand grip force may be relevant when forearm recovery is a limiting consideration. The results do not support presenting contrast therapy as an immediate strength-restoration intervention because hand grip strength did not separate meaningfully between groups immediately after fatigue or at 24 h.

The findings also do not establish that the intervention improves climbing grade, time to exhaustion on a fingerboard, movement quality, injury prevention, or readiness to return after injury. Minimal clinically important differences have not been established for the present microvascular, stiffness, pressure-pain-threshold, or TQR outcomes in climbers. Consequently, statistical and standardized effect-size magnitudes should not be equated automatically with clinically meaningful benefits.

Implementation should also be considered. The intervention comprised 27 supervised sessions over 8 weeks and combined alternating temperatures with pneumatic compression. Its practical value will depend on treatment availability, athlete tolerance, session burden, cost, and whether similar effects can be obtained with fewer sessions or simpler recovery strategies. The most defensible current application is as an adjunct following selected high-load forearm sessions, rather than as a routine substitute for adequate recovery time, sleep, nutrition, and appropriate training-load progression.

## 5. Conclusions

In amateur climbers exposed to a fixed climbing-specific forearm-loading protocol that standardized task structure but not relative finger-flexor intensity, repeated contrast therapy was associated with more favorable recovery than passive rest across microcirculatory, biomechanical, nociceptive, functional, and perceptual outcomes, while biochemical effects were limited and transient. The intervention did not meaningfully alter the immediate acute fatigue response, but it improved 24 h and 48 h recovery patterns, enhanced post-occlusive reactive hyperemia after 8 weeks, reduced forearm stiffness, increased pressure pain threshold, and supported later restoration of maximal hand grip strength. These findings indicate that contrast therapy was associated with more favorable cutaneous perfusion, PORH-derived, and neuromechanical recovery outcomes after forearm loading. However, the study did not establish the underlying vascular mechanism, and the results should not be interpreted as evidence of enhanced nitric oxide production, NO-dependent vasodilation, or endothelial function. Interpretation is further constrained by the passive comparator, the cutaneous surrogate measurement, the absence of direct climbing-performance outcomes, and the lack of participant-specific relative-intensity standardization during the fatigue protocol.

## Figures and Tables

**Table 1:** Participant characteristics and vascular safety screening values by randomized group.

Variable	Passive Recovery Group (n = 20)	Contrast Therapy Group (n = 20)
Female sex, n (%)	7 (35)	8 (40)
Age (years)	30.7 ± 5.1	29.4 ± 6.1
Body height (cm)	174.7 ± 8.8	175.1 ± 9.2
Body mass (kg)	73.3 ± 9.7	70.7 ± 9.9
Body mass index (kg/m^2^)	24.0 ± 2.4	23.0 ± 2.6
Climbing experience (years)	5.5 ± 2.1	6.2 ± 2.3
Right-dominant upper limb, n (%)	18 (90)	18 (90)
Systolic blood pressure (mmHg)	126.6 ± 6.2	126.7 ± 4.6
Diastolic blood pressure (mmHg)	80.1 ± 5.2	79.2 ± 5.1
Resting heart rate (beats/min)	72.1 ± 8.0	74.2 ± 5.5
Ankle–brachial index	1.04 ± 0.03	1.04 ± 0.03
100% arterial occlusion pressure (mmHg)	183.8 ± 7.0	184.8 ± 8.0

Values are mean ± standard deviation unless otherwise indicated. Categorical variables are presented as n (%). Baseline characteristics are reported descriptively by randomized group; no formal between-group hypothesis tests were performed.

**Table 2:** Descriptive primary outcome values across recovery time points.

Outcome	Time Point	Passive Recovery Group (n = 20)	Contrast Therapy Group (n = 20)
Resting tissue perfusion (PU), initial acute session	Rest	18.75 ± 0.86	18.33 ± 0.81
Resting tissue perfusion (PU), initial acute session	Immediately after fatigue	21.51 ± 1.02	21.08 ± 1.04
Resting tissue perfusion (PU), initial acute session	24 h after fatigue	18.78 ± 0.94	19.70 ± 0.88
Resting tissue perfusion (PU), initial acute session	48 h after fatigue	18.75 ± 0.89	19.73 ± 0.84
Resting tissue perfusion (PU), after 8-week intervention	Rest	18.82 ± 0.88	19.18 ± 0.83
Resting tissue perfusion (PU), after 8-week intervention	Immediately after fatigue	21.69 ± 1.05	22.07 ± 0.89
Resting tissue perfusion (PU), after 8-week intervention	24 h after fatigue	18.93 ± 0.93	20.64 ± 0.91
Resting tissue perfusion (PU), after 8-week intervention	48 h after fatigue	18.91 ± 0.93	20.61 ± 0.90
Mean forearm muscle stiffness (N/m)	Baseline	346.1 ± 8.8	348.8 ± 7.5
Mean forearm muscle stiffness (N/m)	Post-fatigue	483.8 ± 13.2	487.1 ± 12.5
Mean forearm muscle stiffness (N/m)	24 h	454.6 ± 12.1	426.5 ± 12.3
Mean forearm muscle stiffness (N/m)	48 h	420.6 ± 10.6	351.7 ± 9.6
Mean forearm muscle stiffness (N/m)	Week 8	345.4 ± 10.9	326.2 ± 7.3
Mean pressure pain threshold (N/cm^2^)	Baseline	85.6 ± 5.5	84.4 ± 4.7
Mean pressure pain threshold (N/cm^2^)	Post-fatigue	77.5 ± 5.2	76.5 ± 4.8
Mean pressure pain threshold (N/cm^2^)	24 h	84.8 ± 5.3	89.2 ± 5.0
Mean pressure pain threshold (N/cm^2^)	48 h	86.2 ± 5.7	90.8 ± 5.3
Mean pressure pain threshold (N/cm^2^)	Week 8	85.4 ± 5.6	92.2 ± 5.5
Maximum hand grip strength (kgf)	Baseline	62.1 ± 4.6	61.5 ± 6.2
Maximum hand grip strength (kgf)	Post-fatigue	44.3 ± 3.6	43.1 ± 5.0
Maximum hand grip strength (kgf)	24 h	47.0 ± 3.9	46.9 ± 5.5
Maximum hand grip strength (kgf)	48 h	51.6 ± 3.6	58.9 ± 6.0
Maximum hand grip strength (kgf)	Week 8	62.0 ± 4.8	65.2 ± 6.7

Values are mean ± standard deviation. Resting tissue perfusion is expressed in perfusion units. For muscle stiffness and pressure pain threshold, values are means averaged across the three anatomical forearm measurement points. Baseline maximal hand grip strength was assessed as a functional outcome and was not used to prescribe the fingerboard load. Because hand grip dynamometry was not performed using the same edge and half-crimp configuration, it should not be interpreted as the climbing-specific maximal force against which protocol intensity could be normalized.

**Table 3:** Cutaneous post-occlusive reactive hyperemia outcomes at baseline and week 8.

Outcome	Passive Recovery Baseline	Passive Recovery Week 8	Contrast Therapy Baseline	Contrast Therapy Week 8	Between-Group Difference in Change (95% CI)	Adjusted *p*	Hedges’ g (95% CI); Magnitude
Peak reactive hyperemic perfusion (PU)	52.32 ± 5.75	52.35 ± 5.68	50.53 ± 5.25	56.76 ± 5.91	+6.21 (+5.62 to +6.79)	<0.001	+6.68 (+5.05 to +8.30); Extremely large
Time to peak perfusion (s)	47.3 ± 7.2	47.2 ± 7.4	45.6 ± 6.0	39.9 ± 5.6	−5.6 (−6.2 to −5.1)	<0.001	−6.56 (−8.16 to −4.96); Extremely large
Recovery time to baseline perfusion (s)	270.1 ± 19.4	270.5 ± 19.7	269.2 ± 15.2	235.6 ± 14.7	−34.0 (−36.6 to −31.4)	<0.001	−8.27 (−10.23 to −6.31); Extremely large
Post-occlusive reactive hyperemia index (ratio)	2.80 ± 0.32	2.78 ± 0.31	2.76 ± 0.29	2.96 ± 0.31	+0.21 (+0.17 to +0.25)	<0.001	+3.48 (+2.48 to +4.47); Very large
Resting tissue perfusion (PU)	18.75 ± 0.86	18.82 ± 0.88	18.33 ± 0.81	19.18 ± 0.83	+0.77 (+0.61 to +0.94)	<0.001	+3.00 (+2.09 to +3.92); Very large
Biological zero perfusion (PU)	3.08 ± 0.39	3.10 ± 0.39	2.91 ± 0.44	2.91 ± 0.44	−0.01 (−0.03 to +0.01)	0.287	−0.34 (−0.96 to +0.29); Small

Values are mean ± standard deviation. The between-group difference in change is calculated as contrast therapy change minus passive recovery change. *p*-values are Holm-adjusted within the post-occlusive reactive hyperemia outcome family. PU = perfusion units. PORH outcomes describe the cutaneous perfusion response to transient ischemia and should not be interpreted as direct measurements of nitric oxide bioavailability or endothelial function.

**Table 4:** Between-group contrasts for recovery outcomes.

Outcome	Contrast	Passive Recovery Change	Contrast Therapy Change	Between-Group Difference (95% CI), Observed or Back-Transformed Scale	Adjusted *p*	Hedges’ g on the Analysis Scale (95% CI); Magnitude
Resting tissue perfusion	Initial acute session, immediate post-exercise percent change from rest	14.74 ± 1.18	14.97 ± 1.27	+0.23 (−0.56 to +1.01)	0.559	+0.18 (−0.44 to +0.80); Trivial
Resting tissue perfusion	Initial acute session, 24 h percent change from rest	0.15 ± 1.48	7.43 ± 0.28	+7.28 (+6.58 to +7.98)	<0.001	+6.72 (+5.09 to +8.35); Extremely large
Resting tissue perfusion	Initial acute session, 48 h percent change from rest	0.01 ± 1.44	7.64 ± 0.32	+7.62 (+6.94 to +8.31)	<0.001	+7.14 (+5.42 to +8.87); Extremely large
Resting tissue perfusion	After 8-week intervention, immediate post-exercise percent change from rest	15.20 ± 0.96	15.06 ± 1.18	−0.15 (−0.84 to +0.54)	0.668	−0.13 (−0.75 to +0.49); Trivial
Resting tissue perfusion	After 8-week intervention, 24 h percent change from rest	0.54 ± 1.68	7.61 ± 0.31	+7.07 (+6.28 to +7.87)	<0.001	+5.72 (+4.30 to +7.15); Extremely large
Resting tissue perfusion	After 8-week intervention, 48 h percent change from rest	0.46 ± 1.32	7.47 ± 0.31	+7.01 (+6.38 to +7.64)	<0.001	+7.17 (+5.45 to +8.90); Extremely large
Mean forearm muscle stiffness	PostFatigue	+137.7 ± 6.9	+138.3 ± 7.4	+0.5 (−4.1 to +5.1)	0.813	+0.07 (−0.55 to +0.69); Trivial
Mean forearm muscle stiffness	24 h	+108.5 ± 6.1	+77.6 ± 7.1	−30.9 (−35.1 to −26.7)	<0.001	−4.61 (−5.81 to −3.40); Extremely large
Mean forearm muscle stiffness	48 h	+74.6 ± 5.6	+2.9 ± 6.6	−71.7 (−75.6 to −67.8)	<0.001	−11.48 (−14.13 to −8.83); Extremely large
Mean forearm muscle stiffness	Week 8	−0.7 ± 3.8	−22.6 ± 1.7	−22.0 (−23.9 to −20.0)	<0.001	−7.31 (−9.06 to −5.55); Extremely large
Mean pressure pain threshold	PostFatigue	−8.2 ± 1.0	−7.9 ± 1.3	+0.3 (−0.5 to +1.0)	0.495	+0.21 (−0.41 to +0.84); Small
Mean pressure pain threshold	24 h	−0.8 ± 0.9	+4.8 ± 0.8	+5.6 (+5.0 to +6.1)	<0.001	+6.25 (+4.71 to +7.79); Extremely large
Mean pressure pain threshold	48 h	+0.6 ± 0.9	+6.4 ± 1.1	+5.8 (+5.1 to +6.5)	<0.001	+5.51 (+4.13 to +6.90); Extremely large
Mean pressure pain threshold	Week 8	−0.2 ± 1.2	+7.8 ± 1.1	+8.1 (+7.3 to +8.8)	<0.001	+7.01 (+5.32 to +8.71); Extremely large
Maximum hand grip strength	PostFatigue	−17.8 ± 2.4	−18.5 ± 2.7	−0.6 (−2.3 to +1.0)	0.862	−0.25 (−0.87 to +0.38); Small
Maximum hand grip strength	24 h	−15.1 ± 2.5	−14.6 ± 2.7	+0.5 (−1.2 to +2.1)	0.862	+0.18 (−0.44 to +0.80); Trivial
Maximum hand grip strength	48 h	−10.4 ± 1.0	−2.6 ± 0.3	+7.8 (+7.3 to +8.3)	<0.001	+9.95 (+7.63 to +12.27); Extremely large
Maximum hand grip strength	Week 8	−0.1 ± 1.0	+3.7 ± 0.6	+3.8 (+3.2 to +4.3)	<0.001	+4.40 (+3.24 to +5.57); Extremely large
Perceived fatigue	24 h	+3.2 ± 0.6	+2.3 ± 0.8	−1.0 (−1.4 to −0.5)	<0.001	−1.29 (−1.97 to −0.60); Large
Perceived fatigue	48 h	+1.2 ± 0.6	+0.6 ± 0.6	−0.7 (−1.0 to −0.3)	0.004	−1.03 (−1.69 to −0.37); Moderate
Perceived fatigue	Week 8	+0.5 ± 0.6	−0.6 ± 0.7	−1.1 (−1.5 to −0.6)	<0.001	−1.60 (−2.32 to −0.88); Large
Total Quality Recovery	24 h	−4.8 ± 0.9	−3.6 ± 1.0	+1.1 (+0.6 to +1.7)	<0.001	+1.22 (+0.54 to +1.90); Large
Total Quality Recovery	48 h	−2.2 ± 0.7	+0.1 ± 1.1	+2.3 (+1.7 to +2.9)	<0.001	+2.43 (+1.60 to +3.26); Very large
Total Quality Recovery	Week 8	−1.1 ± 0.9	+0.8 ± 0.9	+2.0 (+1.4 to +2.6)	<0.001	+2.17 (+1.38 to +2.96); Very large
Creatine kinase	24 h, ln(follow-up/baseline)	+27.3%	+19.2%	−6.3% (−6.9% to −5.8%)	<0.001	−7.10 (−8.81 to −5.39); Extremely large
Creatine kinase	48 h, ln(follow-up/baseline)	+33.3%	+19.8%	−10.1% (−10.6% to −9.7%)	<0.001	−13.56 (−16.67 to −10.45); Extremely large
Creatine kinase	Week 8, ln(follow-up/baseline)	−0.2%	−0.5%	−0.3% (−1.8% to +1.2%)	1.000	−0.13 (−0.75 to +0.49); Trivial
Interleukin-6	24 h, ln(follow-up/baseline)	+74.8%	+31.6%	−24.7% (−27.5% to −21.8%)	<0.001	−4.78 (−6.01 to −3.54); Extremely large
Interleukin-6	48 h, ln(follow-up/baseline)	+10.7%	+9.8%	−0.8% (−4.1% to +2.6%)	1.000	−0.16 (−0.78 to +0.46); Trivial
Interleukin-6	Week 8, ln(follow-up/baseline)	+3.7%	+5.4%	+1.6% (−6.2% to +10.1%)	1.000	+0.13 (−0.49 to +0.75); Trivial

Changes are calculated relative to the relevant baseline resting value. For resting tissue perfusion, changes are percent changes from the resting value within the same acute testing block and differences are expressed in percentage points. For creatine kinase and interleukin-6, changes are natural-log changes from baseline and are displayed as percentage relative changes. Inference was performed on the log scale. Adjusted *p*-values are Holm-adjusted within the outcome family. The between-group difference is contrast therapy minus passive recovery.

## Data Availability

The datasets used and analyzed during the current study are available from the corresponding author on reasonable request.

## Abbreviations

The following abbreviations are used in this manuscript:

PRG	Passive recovery group
CTG	Contrast therapy group
RPE	Rate of perceived exertion
CK	Creatine Kinase
